# Evaluating prognostic value and stage migration effects using a positive lymph node ratio in adenocarcinoma of the esophagogastric junction

**DOI:** 10.1186/s12885-023-10689-6

**Published:** 2023-03-08

**Authors:** Hajime Kamiya, Shuhei Komatsu, Keiji Nishibeppu, Takuma Ohashi, Hirotaka Konishi, Atsushi Shiozaki, Takeshi Kubota, Hitoshi Fujiwara, Kazuma Okamoto, Eigo Otsuji

**Affiliations:** Division of Digestive Surgery (Gastric Surgery Division), Department of Surgery, Kyoto Prefectural University of Medicine, 465 Kajii-cho, Kawaramachihirokoji, Kamigyo-ku, 602- 8566 Kyoto, Japan

**Keywords:** Positive lymph node ratio, Adenocarcinoma of the esophagogastric junction, Retrieved lymph nodes, Prognosis, Stage migration effect

## Abstract

**Background:**

Adenocarcinoma of the esophagogastric junction (AEG) is increasing worldwide. Lymph node metastasis is an important clinical issue in AEG patients. This study investigated the usefulness of a positive lymph node ratio (PLNR) to stratify prognosis and evaluate stage migration.

**Methods:**

We retrospectively analysed 117 consecutive AEG patients (Siewert type I or II) who received a lymphadenectomy between 2000 and 2016.

**Results:**

A PLNR cut-off value of 0.1 most effectively stratified patient prognosis into two groups (*P* < 0.001). Also, prognosis could be clearly stratified into four groups: PLNR = 0, 0 < PLNR < 0.1, 0.1 ≤ PLNR < 0.2, and 0.2 ≤ PLNR (*P* < 0.001, 5-year survival rates (88.6%, 61.1%, 34.3%, 10.7%)). A PLNR ≥ 0.1 significantly correlated with tumour diameter ≥ 4 cm (*P* < 0.001), tumour depth (*P* < 0.001), greater pathological N-status (*P* < 0.001), greater pathological Stage (*P* < 0.001), and oesophageal invasion length ≥ 2 cm (*P* = 0.002). A PLNR ≥ 0.1 was a poor independent prognostic factor (hazard ratio 6.47, *P* < 0.001). The PLNR could stratify prognosis if at least 11 lymph nodes were retrieved. A 0.2 PLNR cut-off value discriminated a stage migration effect in pN3 and pStage IV (*P* = 0.041, *P* = 0.015) patients; PLNR ≥ 0.2 might potentially diagnose a worse prognosis and need meticulous follow-up post-surgery.

**Conclusion:**

Using PLNR, we can evaluate the prognosis and detect higher malignant cases who need meticulous treatments and follow-up in the same pStage.

**Supplementary Information:**

The online version contains supplementary material available at 10.1186/s12885-023-10689-6.

## Background

Recent advances in diagnostic processes, treatment invasiveness, surgical techniques, perioperative management, and chemoradiotherapy have improved the early and long-time outcomes of oesophageal and gastric cancers [1, 2]. Moreover, the eradication of *Helicobacter pylori* infection has also significantly contributed to decreasing the incidence of gastric cancer [3, 4]. However, the resulting gastroesophageal reflux disease caused by the eradication of *Helicobacter pylori* (among various other factors) might potentially lead to an increase in the incidence of adenocarcinoma of the esophagogastric junction (AEG) worldwide [5]. Indeed, Siewert type II, which arises from the epithelium of the cardia or short segments of intestinal metaplasia at the esophagogastric junction, has doubled over the past decade [5, 6]. Although the eighth edition of the AJCC/UICC TNM classification system [7] indicates that both Siewert type I and II are classified as oesophageal cancers [8], various clinical issues need to be resolved [2]. Specifically, the appropriate surgical fields for lymph node dissection [9, 10] and lymph node staging systems remain controversial.

A positive lymph node ratio (PLNR), obtained by dividing the metastatic lymph node counts by the retrieved lymph node counts, is reportedly a promising prognostic indicator of various cancers [11–14]. In this study, we hypothesized that a PLNR could be a promising prognostic factor and provide a staging system to stratify prognoses and reflect the stage migration effects of AEG. The results of our study may provide evidence that a PLNR could be a better system for predicting prognosis and a potential indicator of stage migration effects.

## Methods

### Patients and procedures

This study was institutionally approved by the Kyoto Prefectural University of Medicine, and each participant provided written informed consent. A total of 117 patients who underwent curative surgery for AEG, classified as Siewert type I or II, at our institute between 2000 and 2016 were included in this study. We precisely defined Siewert type based on pathological mapping and macroscopic measurements of the distance between the tumour epicentre and the esophagogastric junction. Furthermore, we retrospectively analysed clinicopathological features and prognostic outcomes. Finally, we evaluated the compatibility of our findings with the eighth edition of the AJCC/UICC TNM classification system for AEG [7, 15].

The postoperative follow-up program at our institution comprises a regular physical examination as well as laboratory blood tests and chest X-rays every three or six months. Endoscopy and ultrasonography, or computed tomography, were performed annually for the first five years, if possible. All enrolled patients underwent pathological or macroscopic curative resection (R0). Histological types were classified as differentiated (papillary adenocarcinoma, or moderately or well-differentiated adenocarcinoma) or undifferentiated (poorly differentiated or undifferentiated adenocarcinoma, signet-ring cell carcinoma, or mucinous adenocarcinoma) based on the 15th edition of the Japanese Classification of Gastric Carcinoma [16]. Patients with bulky metastatic lymph nodes underwent neoadjuvant chemotherapy (NAC). The regimen of NAC was S-1 and cisplatin according to Japanese gastric cancer guidelines [16]. Patients who underwent NAC were 10.2% (12/117) of all patients. Patients with pStage II or high underwent postoperative S-1 adjuvant chemotherapy for one year according to the ACTS-GC (Adjuvant Chemotherapy Trial of TS-1 for Gastric Cancer) study [17].

### Assessment of the clinical impact of the positive lymph node ratio (PLNR)

**Fig. 1:**
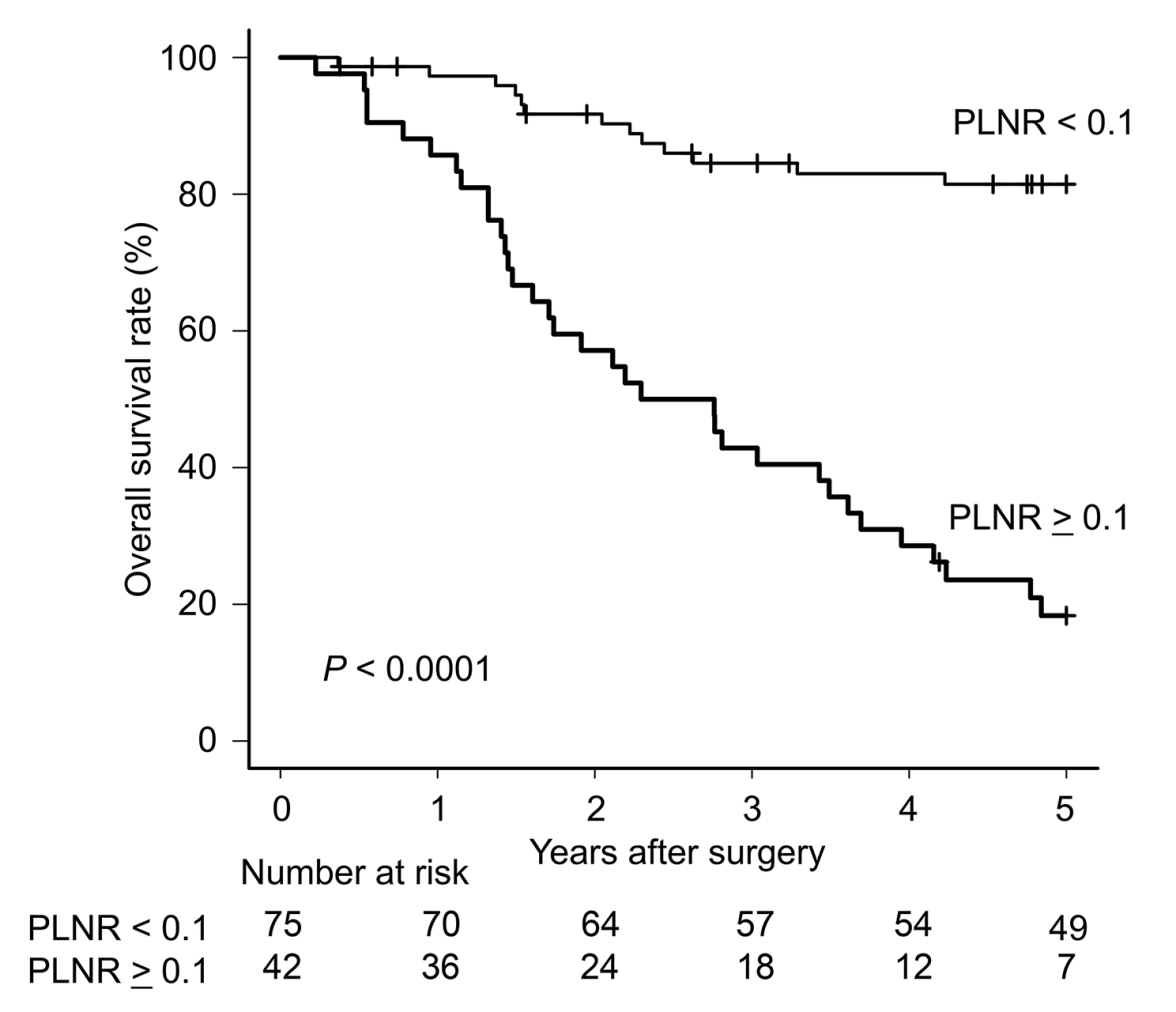
Survival curves for AEG patients according to the PLNR. A PLNR cut-off value of 0.1 most significantly stratified the prognosis of AEG patients into two groups (P < 0.0001, 5-year survival rate; PLNR < 0.1 vs. PLNR ≥ 0.1: 79.7% vs. 18.3％)

**Fig. 2:**
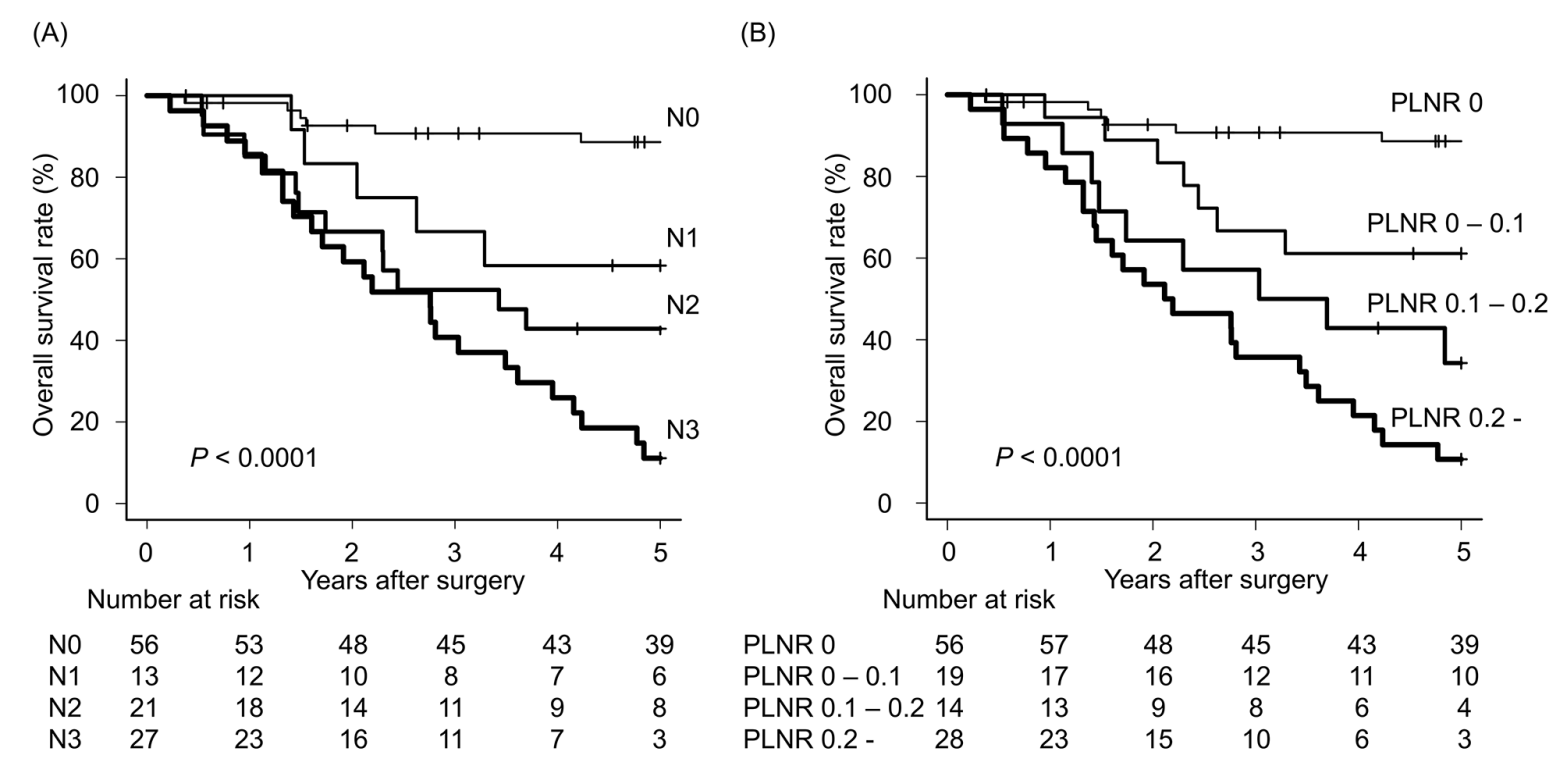
Comparison of the survival curves of AEG patients according to the N classification of the TNM staging system and the PLNR. **A**. By using the N classification of TNM staging, the prognoses of N2 and N3 patients were very similar until 2.5 years after surgery. The 2.5-year survival rates of patients with N0, N1, N2 and N3 were 90.7%, 75.0％, 52.4％ and 51.9％, respectively. **B**. By using the PLNR cut-off values (PLNR 0, PLNR 0 - < 0.1, PLNR 0.1 - < 0.2 and PLNR ≥ 0.2), patients were more appropriately classified into four groups compared to using the N classification of TNM staging. The 2.5-year survival rates of patients with PLNR 0, PLNR 0 - < 0.1, PLNR 0.1 - < 0.2 and PLNR ≥ 0.2 were 90.7％, 66.7％, 57.1％ and 35.7％, respectively

**Fig. 3:**
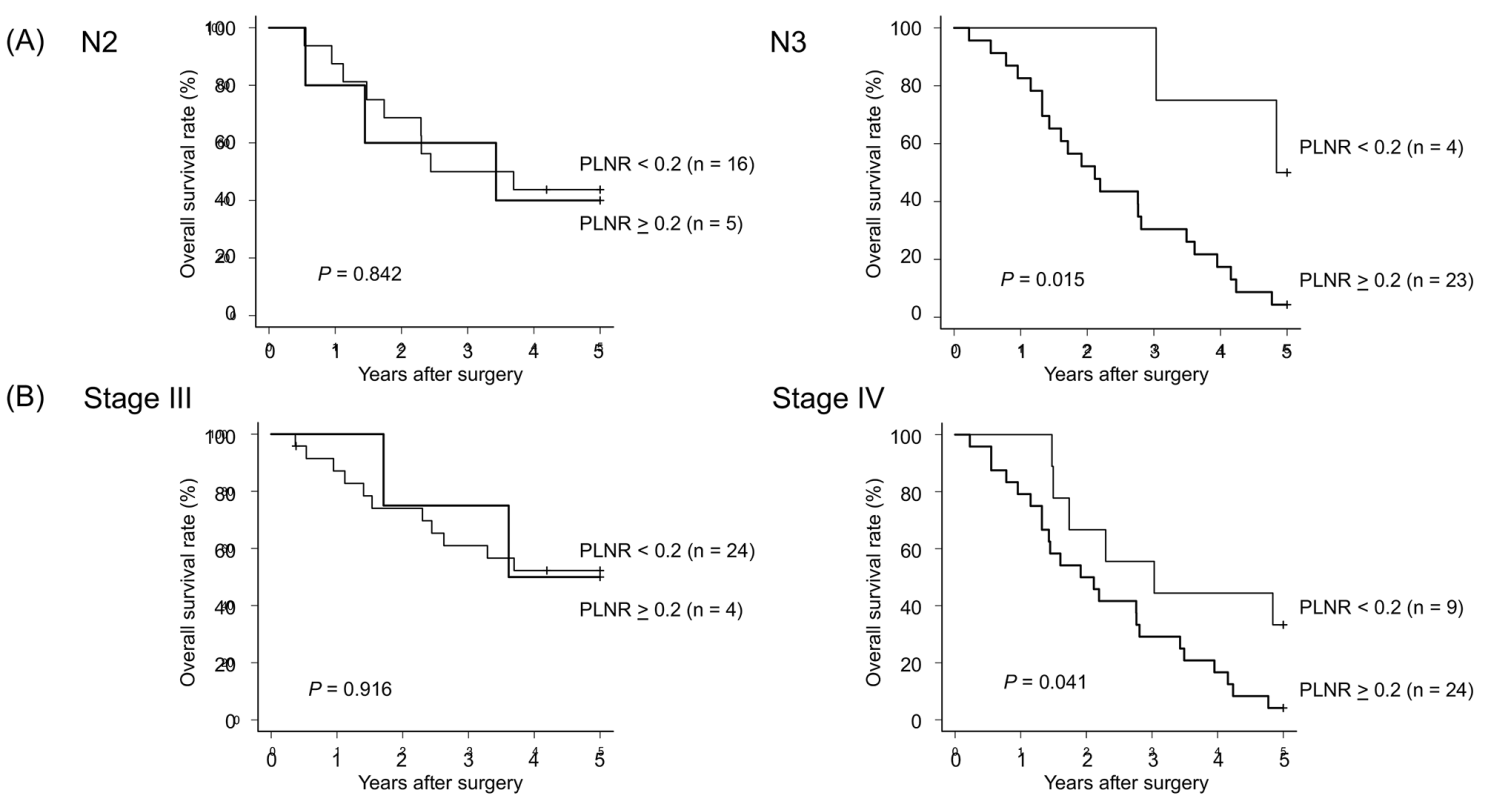
Evaluation of the PLNR stratification of prognosis and detection of stage migration effects according to a PLNR cut-off value of 0.2. The cut-off value of 0.2 detected patients with stage migration effects in pN3 and pStage IV. **A**. pN3 patients with a PLNR ≥ 0.2 (n = 23) had a poorer prognosis than pN3 patients with a PLNR < 0.2 (n = 4). Moreover, pN3 patients with a PLNR < 0.2 had a similar prognosis to pN2 patients (n = 21) (5-year survival rate; pN3 PLNR < 0.2 vs. pN2 vs. pN3 PLNR ≥ 0.2: 50.0% vs. 42.9% vs. 4.3％). **B**. pStage IV patients with a PLNR ≥ 0.2 (n = 24) had a poorer prognosis than pStage IV patients with a PLNR < 0.2 (n = 9). Moreover, pStage IV patients with a PLNR < 0.2 had a similar prognosis to pStage III patients (n = 28) (4-year survival rate; pStage IV-PLNR < 0.2 vs. pStage III: 44.4％ vs. 51.9%)

As previously reported[12], to confirm the clinical feasibility of the PLNR, we firstly investigated whether the average number of retrieved lymph nodes was sufficient in all stages in our cohort (Supplementary Table S1). Secondly, the PLNR was calculated by dividing the total number of pathological metastatic lymph nodes by the total number of retrieved lymph nodes. We calculated the PLNR and performed survival analysis using various cut-off values to compare with the N classification of TNM staging (Supplementary Table S2 and Fig. 1). Thirdly, we investigated differences in prognosis and the related clinicopathological factors according to the PLNR cut-off values (Table 1; Fig. 2) by performing multivariate analysis using Cox’s proportional hazard model (Table 2). Fourth, we examined whether the PLNR could detect stage migration effects following surgery in a single institute (Fig. 3). Finally, we evaluated the impact of the number of retrieved lymph nodes when using the PLNR system (Table 3).

**Table 1:** Comparison of clinicopathological factors between patients with PLNR < 0.1 and PLNR ≥ 0.1

		PLNR < 0.1		PLNR ≥ 0.1		Univariate
		n = 75		n = 42		*P*-value
Age (years)	< 70	42	(56%)	26	(62%)	0.564
	≥ 70	33	(44%)	16	(38%)	
Sex	Female	15	(20%)	5	(12%)	0.315
	Male	60	(80%)	37	(88%)	
Histological type	Differentiated	49	(65%)	24	(57%)	0.429
	Undifferentiated	26	(35%)	18	(43%)	
Venous invasion	Negative	43	(57%)	2	(5%)	**< 0.001**
	Positive	32	(43%)	40	(95%)	
Lymphatic invasion	Negative	43	(57%)	8	(19%)	**< 0.001**
	Positive	32	(43%)	34	(81%)	
Tumour size (mm)	< 40	38	(51%)	6	(14%)	**< 0.001**
	≥ 40	37	(49%)	36	(86%)	
Neoadjuvant chemotherapy	Absence	71	(95%)	34	(81%)	**0.027**
	Presence	4	(5%)	8	(19%)	
Pathological T status	T1	36	(48%)	0	(0%)	**< 0.001**
	T2	11	(15%)	5	(12%)	
	T3	21	(28%)	19	(45%)	
	T4	7	(9%)	18	(43%)	
Pathological N status	N0	56	(75%)	0	(0%)	**< 0.001**
	N1	12	(16%)	1	(2%)	
	N2	6	(8%)	15	(36%)	
	N3	1	(1%)	26	(62%)	
TNM pStage	I	37	(49%)	0	(0%)	**< 0.001**
	II	19	(25%)	0	(0%)	
	III	17	(23%)	11	(26%)	
	IV	2	(3%)	31	(74%)	

**Table 2:** Univariate and multivariate survival analyses using Cox’s proportional hazard model

	Variables			Univariate^a^		Multivariate^b^	
				*P*-value	HR^c^	95% CI^d^	*P*-value
Age (years)	< 70	vs	≥ 70	0.571		-	
Sex	Female	vs	Male	0.524		-	
Histology type	Differentiated	vs	Undifferentiated	0.468		-	
Tumour size (cm)	< 40	vs	≥ 40	< 0.001		-	
Venous invasion	Negative	vs	Positive	< 0.001		-	
Lymphatic invasion	Negative	vs	Positive	< 0.001		-	
Pathological T status	T1-2	vs	T3-4	< 0.001		-	
Pathological N status	N0-1	vs	N2-3	< 0.001		-	
PLNR^e^	< 0.1	vs	≥ 0.1	**< 0.001**	6.48	3.85-12.0	**< 0.001**

^a^ Kaplan-Meier method; significance was determined by log-rank test.

^b^ Multivariate survival analysis was performed using Cox’s proportional hazard model.

^c^ HR: Hazard ratio

^d^ CI: Confidence interval

^e^ PLNR: Positive lymph node ratio

**Table 3:** Effect of the number of retrieved lymph nodes for using the 0.1 PLNR cut-off value

Number of retrieved lymph nodes	%	*P-*value Log-rank test
≥ 16	82.9%	< 0.001
≤ 15	17.1%	0.018
≤ 14	17.1%	0.018
≤ 13	12.0%	< 0.001
≤ 12	6.8%	0.014
≤ 11	4.3%	0.046
≤ 10	3.4%	0.083
≤ 9	2.6%	NE
≤ 8	2.6%	NE
≤ 7	2.6%	NE
≤ 6	2.6%	NE

NE: Not evaluated.

### Statistical analysis

We used EZR (Saitama Medical Center, Jichi Medical University, Saitama, Japan), which is a graphical user interface for R (The R Foundation for Statistical Computing, Vienna, Austria for all analyses. We performed Pearson’s chi-square (χ2) and Fisher’s exact probability tests for categorical variables. For unpaired continuous variables, Student’s t test and Mann-Whitney’s U test were performed to compare clinicopathological characteristics between the two groups. Survival curves were estimated by using the Kaplan-Meier method, and the log-rank test was used to examine statistical differences. The data were stratified for multivariate analysis by using forward and backward stepwise Cox regression methods. A p-value < 0.05 was considered statistically significant [18].

## Results

### Retrieved lymph nodes

Supplementary Table S1 shows the average number of retrieved lymph nodes. The mean number of retrieved lymph nodes was 31.2 among all patients and 23.0 in pStage I, 29.7 in pStage II, 36.1 in pStage III and 37.0 in pStage IV. The mean number of retrieved lymph nodes was sufficient in all stages of our cohort.

### Survival and clinicopathological factors between patients with PLNR < 0.1 and PLNR ≥   0.1

A PLNR cut-off value of 0.1 provided the best stratification of prognosis into two groups in our cohort (P < 0.0001, 5-year survival rate; PLNR ≥ 0.1 vs. PLNR < 0.1: 81.5% vs. 18.3%; Supplementary Table S2, Fig. 1). Compared with a PLNR < 0.1, patients with a PLNR ≥ 0.1 had a significantly higher incidence of larger tumours (*P* < 0.001), presence of NAC (*P* = 0.027), greater T-status (*P* < 0.001), greater N-status (*P* < 0.001), venous and lymphatic invasion (*P* < 0.001) and greater pStage (*P* < 0.001; Table 1). Multivariate analysis using Cox’s proportional hazard model demonstrated that a PLNR ≥ 0.1 was a poor independent prognostic factor [*P* < 0.0001, HR 6.48 (95% CI: 3.85–12.0)] (Table 2).

### Stratifying prognosis with a PLNR cut-off value

By using PLNR cut-off values (i: PLNR 0, ii: PLNR 0 - < 0.1, iii: PLNR 0.1 - < 0.2 and iv: PLNR ≥ 0.2), patients were more appropriately classified into four groups than when using other cut-off values (Fig. 2). By stratifying prognosis using pathological N-status in the TNM classification system, the 5-year survival rates of patients in stages N0, N1, N2 and N3 were 88.6%, 58.3%, 42.9% and 11.1%, respectively (*P* = 4.97 × 10 ^− 10^). In contrast, the 5-year survival rates of patients with a PLNR of 0, 0 - < 0.1, 0.1 - < 0.2 and ≥ 0.2 were 88.6%, 61.1%, 34.3% and 10.7%, respectively (*P* = 1.22 × 10 ^− 11^), demonstrating better stratification compared to using N-status in the TNM classification system.

### Evaluating stage migration effects using the PLNR

We used PLNR cut-off values of 0.1 or 0.2 to detect stage migration effects. As shown in Supplementary Figures S1-4, PLNR clarified the existence of stage migration effects in the same stage and N-status among advanced AEG patients. Definite stage migration effects were detected in Stage IV and N3 status patients with a PLNR cut-off value of 0.2. Figure 3 shows the survival curves separating N-status and pStage with a PLNR cut-off value of 0.2. In brief, there were no stage migration effects in pN2 (5-year survival rate; pN2 PLNR ≥ 0.2 vs. pN2 PLNR < 0.2: 40.0% vs. 43.8%, *P* = 0.842) and pStage III (5-year survival rate; pStage III-PLNR ≥ 0.2 vs. pStage III-PLNR < 0.2: 52.3% vs. 50.0%, *P* = 0.916). Alternatively, the cut-off value of 0.2 could detect stage migration effects in AEG patients with N3 and pStage IV. As a results, 14.8% (4/27) of N3 patients and 27.2% (9/33) of the pStage IV patients had PLNR < 0.2. The 5-year survival rate of patients with N3-PLNR < 0.2 was 50%, similar to that of pN2, which indicated that patients with N3-PLNR < 0.2 were downstaged to N2. Moreover, the 4-year survival rates of pStage IV-PLNR < 0.2 and pStage III were almost similar (4-year survival rate; pStage IV-PLNR < 0.2 vs. pStage III: 44.4% vs. 51.9%), which also indicated that patients with pStage IV-PLNR < 0.2 were downstaged to pStage III. Thus, a PLNR cut-off value of 0.2 could discriminate stage migration effects in pN3 and pStage IV (*P* = 0.041, *P* = 0.015).

### The required number of retrieved lymph nodes to use the PLNR

To evaluate the minimum number of retrieved lymph nodes to use PLNR, we confirmed whether PLNR could stratify the prognosis with a limited number of retrieved lymph nodes.

Using PLNR cut-off value of 0.1, the prognosis could be stratified if the number of retrieved lymph nodes was at least 11 (Table 3). Therefore, it was suggested that a minimum of 11 retrieved lymph nodes was sufficient to use PLNR system.

## Discussion

Although recent studies have suggested the appropriate surgical fields for curative lymphadenectomy in AEG, a more accurate staging system and the number of retrieved lymph nodes required also remain pivotal issues to be determined [2, 9, 10, 19]. In this study, we clearly demonstrated that the PLNR could be a promising staging system for evaluating prognosis and stage migration, enabling the identification of AEG patients who need meticulous treatments and follow-up. Specifically, patients were more correctly classified into four groups using PLNR scores (PLNR 0, PLNR 0 - < 0.1, PLNR 0.1 - < 0.2 and PLNR ≥ 0.2) than the conventional staging system. Also, the PLNR could stratify prognosis when using at least 11 retrieved lymph nodes; a PLNR ≥ 0.1 could be a strong independent prognostic factor. Moreover, a PLNR of 0.2 significantly discriminated stage migration effects and detected high-risk patients with poor prognosis in the N3 stage and pStage IV. Thus, PLNR could potentially be a more reliable staging system in patients with AEG.

Only a few studies have reported the efficacy of a PLNR and determined the cut-off value in AEG. Zhang et al. reported that PLNR cut-off values of 0.2 and 0.45 could predict prognosis more homogeneously than the TNM system [20]. Also, Xu et al. reported that PLNR cut-off values of 0.125 and 0.425 could stratify the prognosis of AEG patients [21]. Zhang et al. suggested a PLNR of 0.4 as the cut-off value [22]. The multiple PLNR cut-off values in previous studies might indicate the need for further investigation with other cohorts, including a Japanese cohort. In this study, we used a cohort with a sufficient median number of retrieved lymph nodes (31.2) and secured follow-up data. Our findings suggest that PLNR cut-off values of 0.1 and 0.2 could be simpler and more reliable than previous values. We believe that these cut-off values are promising candidates for a PLNR staging system in AEG.

Concerning lymph node retrieval to use the PLNR system, Barbour reported that some AEG patients cannot be adequately staged due to the small number of retrieved lymph nodes after surgery, stating that a minimum of 15 dissected lymph nodes is required to evaluate N-status in conventional AEG staging [19]. Numerous factors such as range of lymphadenectomy, passion to find lymph nodes pathologically and intra-abdominal fat volume could affect the number of retrieved lymph nodes [13, 23, 24]. Because these factors are different for each institution, surgeon, and patient, they can cause stage migration, which result in incorrect patient prognosis and inadequate postoperative treatment. Furthermore, these various inconsistent conditions could lead to international prognostic differences in AEG. In addition, we also evaluated the minimum number of retrieved lymph nodes to use PLNR in AEG. Using PLNR cut-off value of 0.1, the prognosis of AEG patients could be stratified with at least 11 retrieved lymph nodes. Our cohort had a limitation that only 2.5% patients had less than 11 retrieved lymph nodes. Therefore, multi-centre and nationwide studies with larger cohorts are required to identify the optimal retrieved lymph nodes.

A novel finding in our study was that the PLNR could evaluate stage migration effects in N3 and Stage IV in AEG. These stage migration effects in advanced N-status and pStage have not been reported in previous studies about the PLNR in AEG [20–22, 25]. We previously reported that PLNR was useful for evaluating stage migration effects in pStage II and III gastric cancer and the extent of local tumour clearance in pN3 gastric cancer [12, 13]. In the present study, pN3 patients with a PLNR < 0.2 had a better 5-year survival rate than N2 patients (50.0% vs. 42.9%), and N3 patients with a PLNR ≥ 0.2 had a very poor prognosis (5-year survival rate 4.3%). Similarly, the prognosis of Stage IV patients with a PLNR ≥ 0.2 was extremely poor compared to those with a PLNR < 0.2 (33.3% vs. 4.2%), which highlighted the importance of distinguishing between patients of the same stage. Also, in AEG, PLNR could be useful for more accurately predicting prognosis, surgical quality checking, and determining postoperative treatment strategies in pN3 and pStage IV patients.

The present study was limited by its retrospective design with a small cohort. Additionally, the prolonged recruitment period over which the analysis was performed at a single institution may have been influenced by possible variations in treatment strategies over time. Multi-centre and nationwide studies using larger cohorts are needed. Moreover, an international study using several large cohorts from Eastern and Western countries is necessary to standardise PLNR cut-off values and improve the efficacy of using a smaller number of retrieved lymph nodes in AEG.

## Conclusion

Using PLNR, we can evaluate the prognosis and detect higher malignant cases who need meticulous treatments and follow-up in the same pStage.

## Electronic supplementary material

Below is the link to the electronic supplementary material.

Supplementary Material 1

## Data Availability

The datasets used during the current study are available from the corresponding author on reasonable request.

## Abbreviations

AEG: Adenocarcinoma of the esophagogastric junction
PLNR: Positive lymph node ratio
