# Supplementary material for: Evaluating prognostic value and stage migration effects using a positive lymph node ratio in adenocarcinoma of the esophagogastric junction
Source: BMC Cancer. 2023 Mar 8;23:218. doi: 10.1186/s12885-023-10689-6 (PMC9996992; doi:10.1186/s12885-023-10689-6)
Supplement: Supplementary file 1 — Supplementary Material 1 [file 12885_2023_10689_MOESM1_ESM.pdf]

Supplemental figure legends

Supplemental Figure S1. Evaluation of the PLNR stratification of prognosis and detection of stage migration effects in each N-status according to a PLNR cut-off value of 0.1.

Supplemental Figure S2. Evaluation of the PLNR stratification of prognosis and detection of stage migration effects in each N-status according to a PLNR cut-off value of 0.2.

Supplemental Figure S3. Evaluation of the PLNR stratification of prognosis and detection of stage migration effects in each pStage according to a PLNR cut-off value of 0.1.

Supplemental Figure S4. Evaluation of the PLNR stratification of prognosis and detection of stage migration effects in each pStage according to a PLNR cut-off value of 0.2.

Supplemental Figure S1

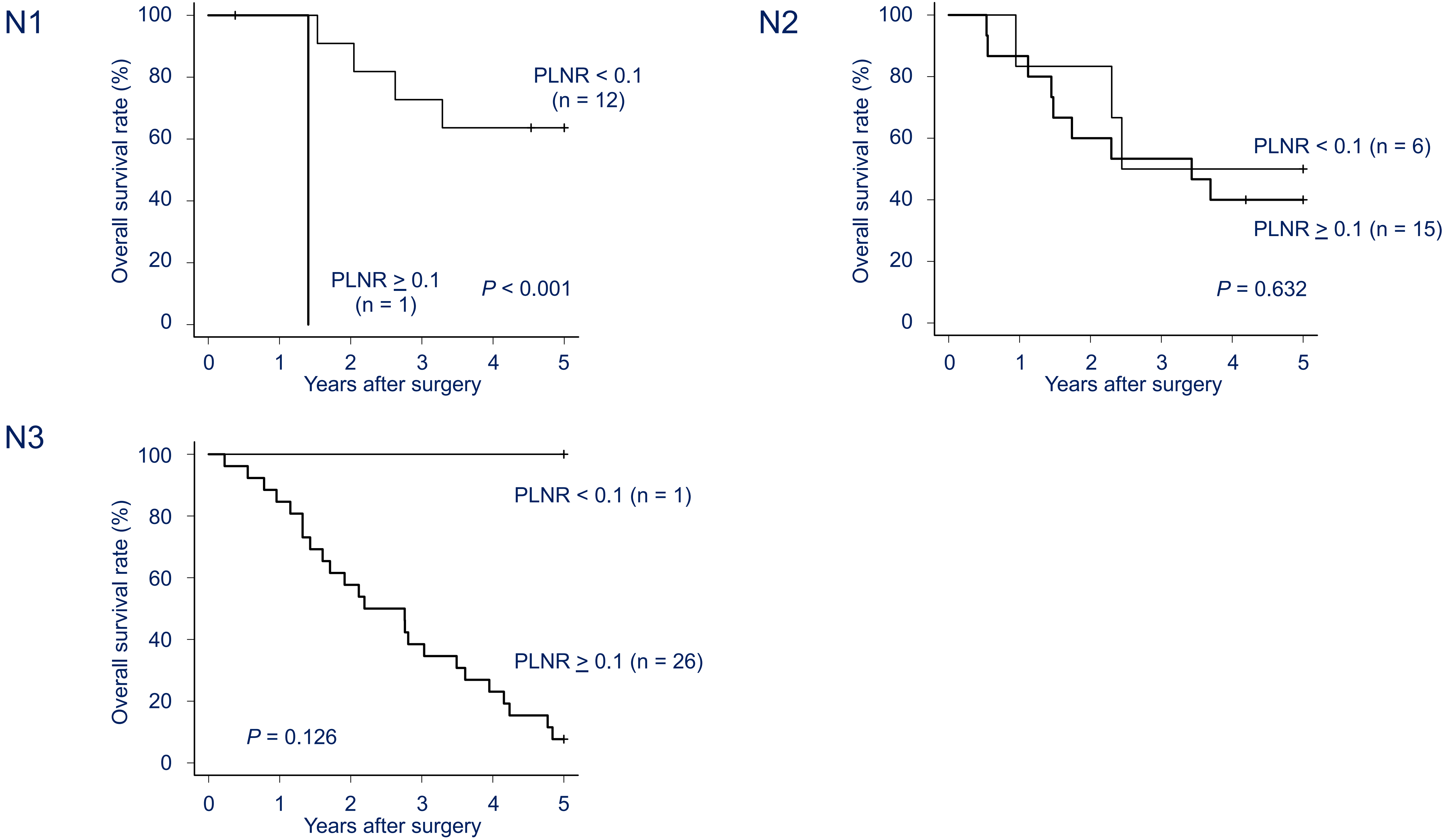

Supplemental Figure S2

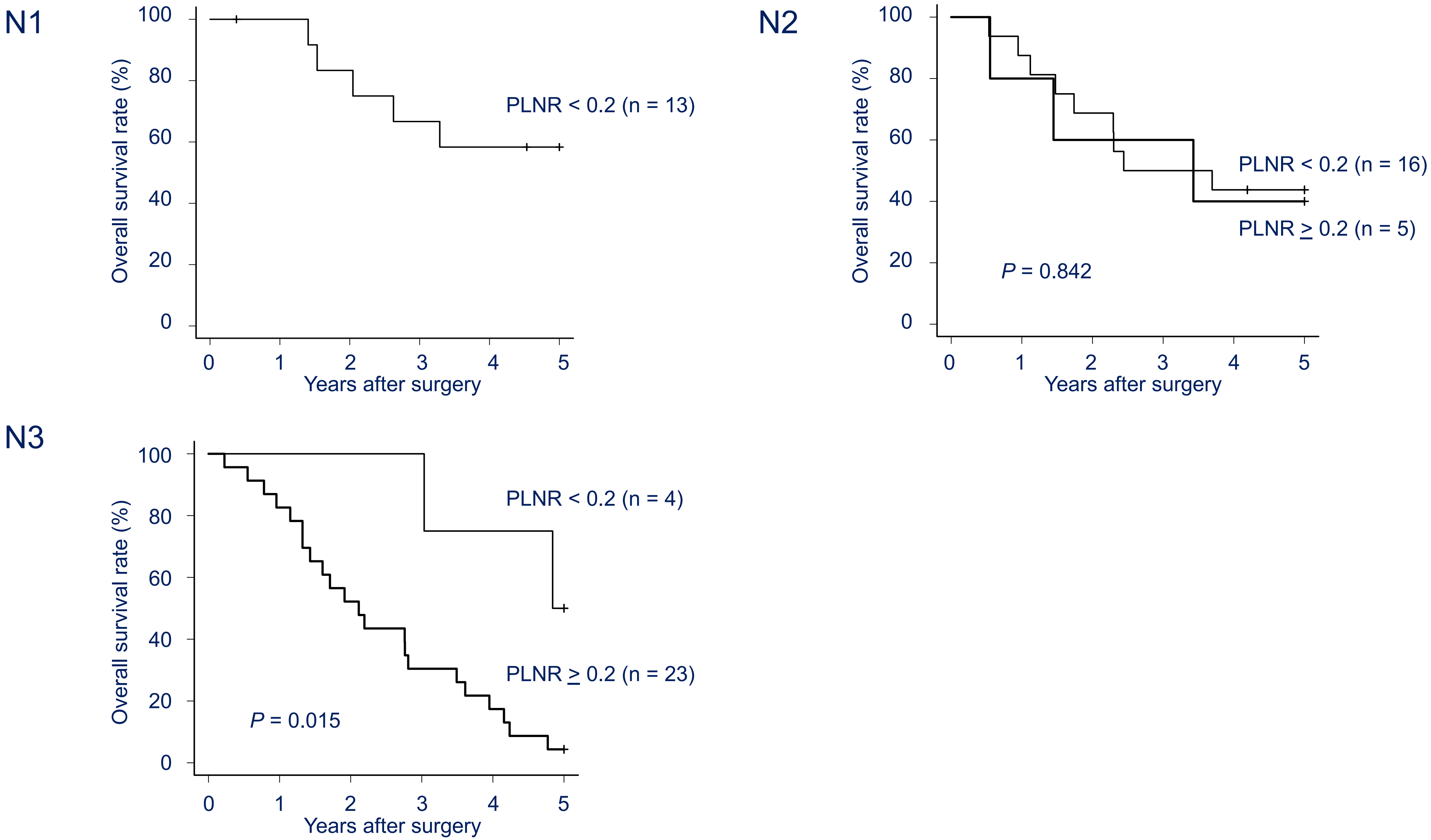

Supplemental Figure S3

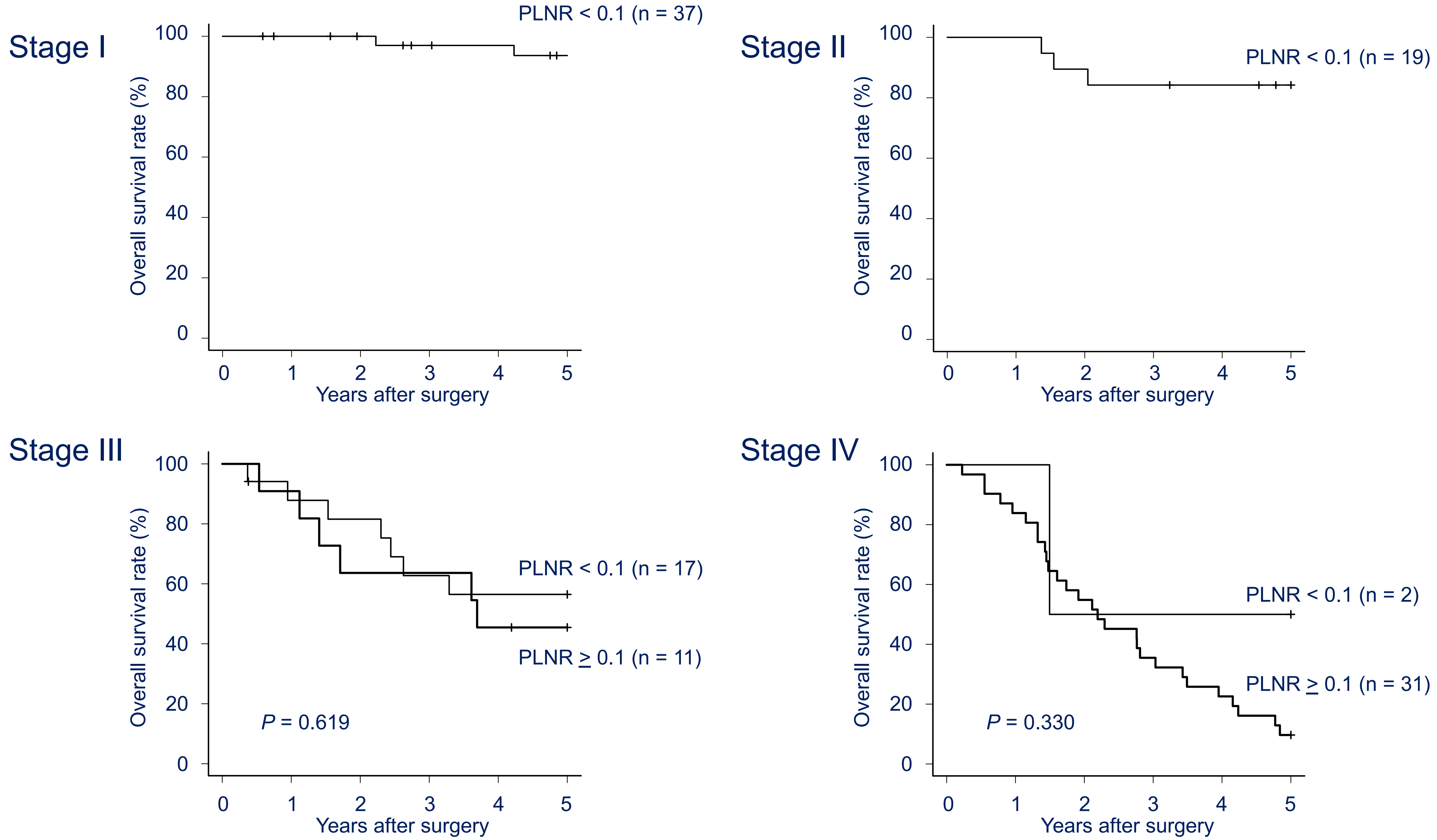

Supplemental Figure S4

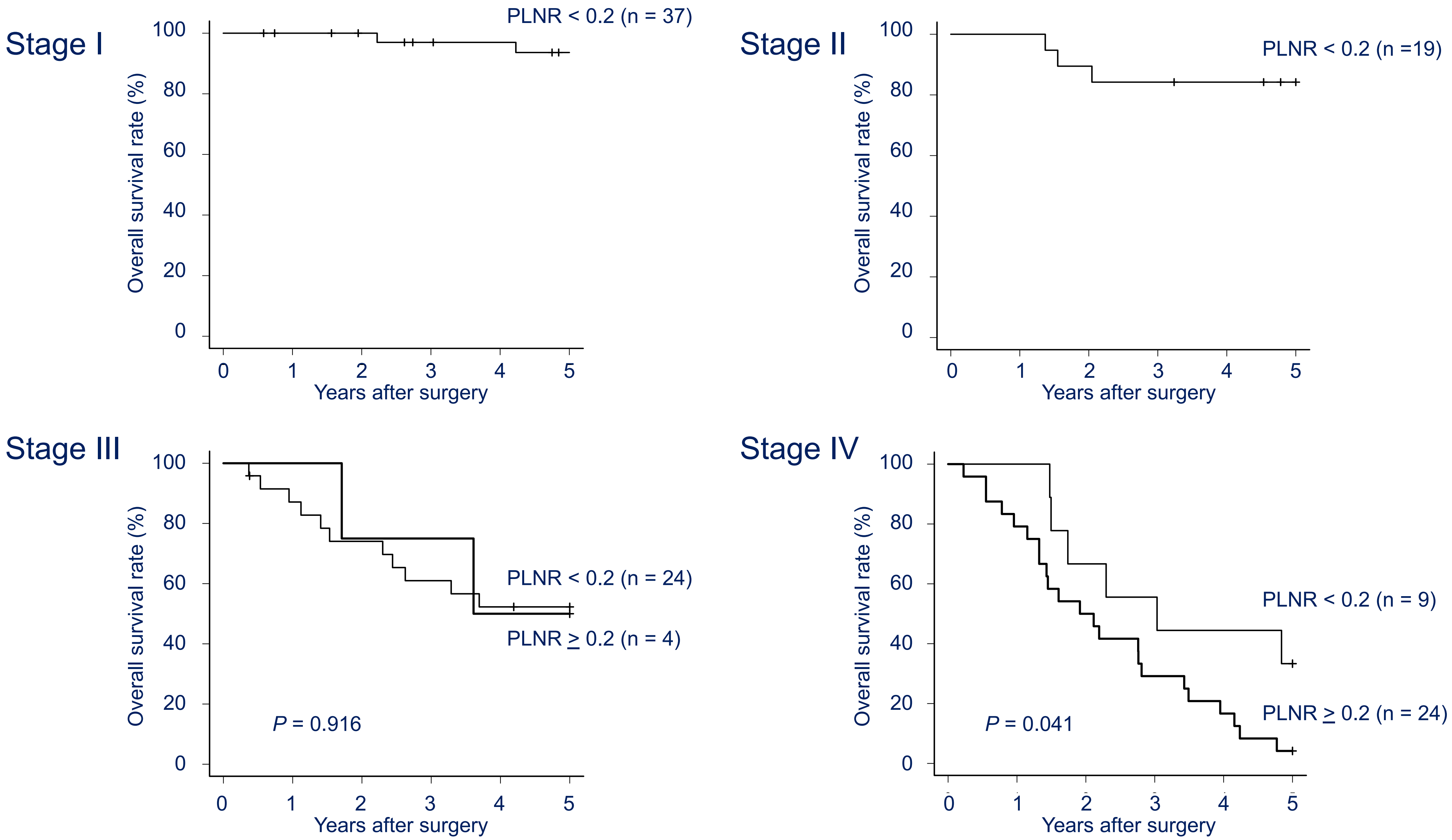

Supplementary Table S1: Average number of retrieved lymph nodes

Stage	N-number	Average number of retrieved lymph nodes
I	37 (31.6%)	23.0
II	19 (16.2%)	29.7
III	28 (23.9%)	36.1
IV	23 (28.2%)	37.0
Total	117 (100%)	31.2

Supplementary Table S2: Survival analysis according to each PLNR cut-off value

PLNR cut-off value			*P*-value (Log-rank test)	
<0.1	vs.	≥ 0.1	1.32×10^{-11}	< 0.0001
<0.2	vs.	≥ 0.2	4.79×10^{-11}	< 0.0001
<0.3	vs.	≥ 0.3	2.99×10^{-9}	< 0.0001
<0.4	vs.	≥ 0.4	1.24×10^{-5}	< 0.0001
<0.5	vs.	≥ 0.5	1.43×10^{-3}	< 0.0001
<0.6	vs.	≥ 0.6	1.43×10^{-3}	< 0.0001
